# Symptoms and patient factors associated with longer time to diagnosis for colorectal cancer: results from a prospective cohort study

**DOI:** 10.1038/bjc.2016.221

**Published:** 2016-08-04

**Authors:** Fiona M Walter, Jon D Emery, Silvia Mendonca, Nicola Hall, Helen C Morris, Katie Mills, Christina Dobson, Clare Bankhead, Margaret Johnson, Gary A Abel, Matthew D Rutter, William Hamilton, Greg P Rubin

**Affiliations:** 1 Primary Care Unit, Department of Public Health and Primary Care, University of Cambridge, Cambridge CB1 8RN, UK; 2 Department of General Practice, University of Melbourne, Melbourne, Australia; 3 School of Medicine, Pharmacy and Health, Durham University, Stockton on Tees TS17 6BH, UK; 4 Nuffield Department of Primary Care Health Sciences, University of Oxford, Oxford OX2 6GG, UK; 5 Lay Member; 6 University Hospital of North Tees, Stockton on Tees TS19 8PE, UK; 7 College House, St Luke's Campus, University of Exeter, Exeter EX2 4TE, UK

**Keywords:** symptoms, diagnosis, diagnostic intervals, time to presentation, delays, help-seeking, stage, colorectal cancer

## Abstract

**Background:**

The objective of this study is to investigate symptoms, clinical factors and socio-demographic factors associated with colorectal cancer (CRC) diagnosis and time to diagnosis.

**Methods:**

Prospective cohort study of participants referred for suspicion of CRC in two English regions. Data were collected using a patient questionnaire, primary care and hospital records. Descriptive and regression analyses examined associations between symptoms and patient factors with total diagnostic interval (TDI), patient interval (PI), health system interval (HSI) and stage.

**Results:**

A total of 2677 (22%) participants responded; after exclusions, 2507 remained. Participants were diagnosed with CRC (6.1%, 56% late stage), other cancers (2.0%) or no cancer (91.9%). Half the cohort had a solitary first symptom (1332, 53.1%); multiple first symptoms were common. In this referred population, rectal bleeding was the only initial symptom more frequent among cancer than non-cancer cases (34.2% *vs* 23.9%, *P*=0.004). There was no evidence of differences in TDI, PI or HSI for those with cancer *vs* non-cancer diagnoses (median TDI CRC 124 *vs* non-cancer 138 days, *P*=0.142). First symptoms associated with shorter TDIs were rectal bleeding, change in bowel habit, ‘feeling different' and fatigue/tiredness. Anxiety, depression and gastro-intestinal co-morbidities were associated with longer HSIs and TDIs. Symptom duration-dependent effects were found for rectal bleeding and change in bowel habit.

**Conclusions:**

Doctors and patients respond less promptly to some symptoms of CRC than others. Healthcare professionals should be vigilant to the possibility of CRC in patients with relevant symptoms and mental health or gastro-intestinal comorbidities.

Colorectal cancer (CRC) is the third most common cancer world wide and the United Kingdom's fourth commonest (Cancer Research UK). Despite the introduction of screening programmes in many countries, most cases present symptomatically. Although 5-year survival rates for CRC have improved to over 60% in the last decade, CRC patients in the United Kingdom continue to have poorer survival rates than those in comparable countries (Coleman *et al*, 2011; Allemani *et al*, 2015). This may partly be due to longer intervals between the onset of cancer symptoms and the patient's presentation to healthcare, compounded by unequal access to optimal diagnostics and specialist care, leading to more late-stage diagnoses (Neal and Allgar, 2005; Maringe *et al*, 2013). There are also gender, age and region differences in time to diagnosis across the United Kingdom (Din *et al*, 2015).

The diagnostic process comprises a series of stages or intervals, each contributing to the overall period of time between onset of symptom/s and initiation of treatment (Weller *et al*, 2012). The total diagnostic interval (TDI) comprises the patient interval (PI – from first symptom onset to first healthcare consultation) and the health system interval (his – from first consultation via referral and investigations to diagnosis and initiation of treatment) (Weller *et al*, 2012): shorter PIs and HSIs are associated with improved CRC prognoses (Torring *et al*, 2011; Neal *et al*, 2015). For CRC, recent evidence suggests that both intervals are longer than for other common cancers such as lung or urological cancer (Dregan *et al*, 2013; Keeble *et al*, 2014; Neal *et al*, 2014a; Lyratzopoulos *et al*, 2015b).

Colorectal cancer patients may be symptomatic for many months before presentation. They may experience multiple symptoms, both bowel-specific (rectal bleeding, change in bowel habit and abdominal pain) and systemic (loss of weight or appetite and fatigue) (Hamilton *et al*, 2005; Rasmussen *et al*, 2015). Not all individuals interpret their initial symptoms as serious and may attribute them to normal variation, ageing, lifestyle or other comorbidities (Macleod *et al*, 2009; Hall *et al*, 2015; McLachlan *et al*, 2015). International comparisons suggest that lower age-related risk and highest perceived barriers to symptomatic presentation were reported in the United Kingdom (Forbes *et al*, 2013); other potential influences include negative beliefs about cancer outcomes (Beeken *et al*, 2011; Lyratzopoulos *et al*, 2015a) and poor awareness of the risk of cancer (Simon *et al*, 2010; Quaife *et al*, 2014).

Once the decision to seek medical help has been made, further delays may occur. One third of CRC patients have three or more consultations with a general practitioner (GP) before referral, compared with only 17.9% for all cancers (Lyratzopoulos *et al*, 2013). Furthermore, the pathway to diagnosis in primary care may be complex, with delays arising when presentation is complicated by comorbid conditions, when referral is delayed or declined, or from false-negative investigation (Rubin *et al*, 2014).

Symptoms of possible CRC much more commonly arise from benign or self-limiting conditions and GPs face a considerable challenge to evaluate patients while making efficient use of hospital-based resources (Hansen *et al*, 2015). Much of the evidence about the predictive value of symptoms for cancer or their association with later diagnosis comes from retrospective studies of people with CRC (Esteva *et al*, 2013) or from general practice data sets (Hamilton *et al*, 2005); these are limited by quality of data recording. Little is known about the diagnostic pathways of those whose symptoms transpire not to be caused by CRC or about which symptoms are associated with less timely diagnosis. We therefore recruited a prospective cohort of patients with symptoms suggestive of CRC at the point of their referral. We aimed to investigate the symptoms, clinical factors and socio-demographic factors associated with CRC diagnosis and time to diagnosis.

## Materials and methods

### Setting and governance

We recruited patients from four secondary care hospitals in the East (*n*=1) and North East of England (*n*=3) between December 2010 and March 2013. We gained all appropriate NHS ethics (reference: 10/H0306/50) and clinical governance approvals. The SYMPTOM colorectal study was conducted alongside the SYMPTOM lung and pancreas studies, collectively part of the NIHR-funded DISCOVERY programme of applied research. The methods of data collection and analysis have already been described with the reporting of the SYMPTOM lung study (Walter *et al*, 2015) and hence will only be outlined briefly here.

### Patient recruitment

All GP referral letters to urgent and routine colorectal and gastrointestinal clinics in participating hospitals were reviewed by a research nurse, to identify patients aged ⩾40 years whose referral mentioned any one or more symptoms from a pre-specified list of symptoms known to be associated with CRC (Supplementary Material). These patients were sent an information sheet, the SYMPTOM colorectal questionnaire and a reply-paid envelope for return. Patients were not eligible for the study if they were already undergoing treatment for any cancer (excluding non-melanotic skin cancer) or had such serious mental and/or physical disease that they were not considered suitable to complete a questionnaire, based on review of the medical record by the research nurse. Patients referred as a result of participation in the Bowel Cancer Screening Programme were excluded. No reminder letters were sent to non-responders, as required by the research ethics committee.

### Data collection

We based our data collection, analysis and reporting on the Aarhus statement for the conduct of cancer diagnostic studies and STROBE guidelines for reporting observational studies (von Elm *et al*, 2007; Weller *et al*, 2012).

#### Patient data

Patient data were collected using the SYMPTOM questionnaire, completed and returned to the research team within 3 months of being sent; most completed it within 2 weeks and before receiving their diagnosis. The SYMPTOM colorectal questionnaire was derived from the previously validated C-SIM questionnaire (Neal *et al*, 2014b). This had been developed for use among people recently diagnosed with cancer and hence we modified it for use before diagnosis, consulting widely with clinical and research colleagues, and patient representatives, to ensure it was worded sensitively, as cancer may not have been explicitly mentioned as a possibility by the referring GP. The questionnaire asked for the first symptom noticed by the participant and then sought the presence or absence of specific symptoms (Supplementary Material). The remaining sections contained items about other symptoms and patient factors, including demographic characteristics and comorbidities.

#### Primary care data

Participants' GPs completed a short proforma from the patient's clinical record, including dates of the first presentation with any symptom from the questionnaire within the previous 2 years, plus its duration, if recorded.

#### Hospital data

We extracted data from hospital medical records on the following: date and type of referral (urgent, routine, emergency and other); date of first specialist consultation; dates of investigations and their findings; date of diagnosis (histological and clinical); and dates of MDT meetings and their clinical decisions. Data extraction took place a minimum of 6 months after recruitment, to allow sufficient time for completion of investigation and initiation of treatment. We undertook double data abstraction of a 5% sample of selected hospital data (dates of referral, first appointment, diagnosis and stage) and confirmed an acceptable level of agreement (>80% for dates, >90% for diagnosis and stage, Cohen's *κ*>0.75 for all), with any discrepancies generally minor.

### Data handling

#### Clinical outcomes

We used the date on the first confirmatory histology report as the date of diagnosis when this was available for both cancer (ICD codes C18-C20) and non-malignant conditions (Weller *et al*, 2012). Otherwise, we used the first date of the clinical diagnosis in the hospital medical record. Participants were classified into three groups: those with primary CRC (CRC), other intra-abdominal cancer (OC) and no cancer (NC). The main analyses focused on CRC *vs* NC, with secondary analyses including all cancers (CRC plus OC) *vs* NC. Colorectal cancer staging was categorised using TNM status at diagnosis (Travis *et al*, 2012) and further categorised into early stage (stages I and II) and late stage (stages III and IV). Complex diagnoses, or cases with incomplete data, were agreed by an adjudication group of clinicians associated with the study (FW, JE, GR and MR).

#### Demographic and clinical variables

Demographic details included the following: gender; age (treated as a continuous variable); ethnicity (coded as White *vs* non-White); smoking status; educational status; occupational status; living alone; and postal code, used to assign groups to quintiles of the Index of Multiple Deprivation (1 ‘least deprived' to 5 ‘most deprived'). Clinical variables relating to comorbidities included the following: gastro-intestinal disease (inflammatory bowel disease, irritable bowel syndrome and peptic ulcer); respiratory disease (chronic obstructive pulmonary disease/asthma/other lung disease), anxiety/depression, heart disease, diabetes and arthritis. Family history of cancer was also identified (present *vs* absent).

#### Symptoms

All symptoms reported up to 2 years before diagnosis were entered into the analysis (symptoms of longer than 2 years duration were unlikely to be associated with the subsequent diagnosis) (Hamilton *et al*, 2005; Biswas *et al*, 2015). Where participants provided estimated dates, these were converted to exact dates by adapting an algorithm used in the C-SIM trial (Neal *et al*, 2014b; Walter *et al*, 2015). We used exact dates where available and estimated dates otherwise. Furthermore, if a participant's unprompted, or free text, first symptom matched their response to a question about specific symptoms, they were given the corresponding symptom code and the earlier date. A first symptom was thus identified for each participant. Many participants reported more than one first symptom, termed ‘multiple first symptoms'. ‘Subsequent symptoms' were defined as any symptom occurring after a first symptom and before diagnosis.

#### The TDI

The TDI, or ‘time to diagnosis', was defined as the time from onset of the first symptom/s to the date of diagnosis. Where the date of first presentation to healthcare was known, the PI, defined as the time from first symptom/s to the first presentation, and the HSI, defined as the time from first presentation to diagnosis, were also calculated.

### Analysis

Descriptive analyses were performed on all demographic, clinical and symptom data for the group as a whole and by diagnostic group (CRC, all cancer (CRC plus OC) and NC). The CRC group was also sub-analysed by stage at diagnosis. The TDI and PI for each group was compared using Wilcoxon rank-sum test.

Clinically relevant demographics, comorbidities, first symptom/s and family history of cancer variables were *a priori* included in multivariate analyses, to identify predictors of time to diagnosis. Variables present in fewer than 10 participants were excluded. We chose to analyse CRC, OC and NC groups together for all outcomes, because at the time of presentation and referral the final diagnosis was unknown. Flexible parametric survival models were used to model the TDI, the PI and the HSI. In these cases, rather than death, the event considered to be ‘failure' in the survival model is either first presentation to healthcare or diagnosis as appropriate. We preferred flexible parametric survival models (Lambert and Royston, 2009) over the Cox model, as they allow the investigation of duration-varying effects. We present results from two models: first, with only time-constant effects and, second, one that allows for duration-varying effects for symptoms. The former model provides average hazard ratios over time for all variables similar to a Cox model and makes it easier to compare the effects of different factors. The second model allows us to examine in more detail how their effect varies with duration. Symptoms with duration-varying effects were identified using a forward selection approach described in Royston and Lambert (2011) and using a significance level of *P*⩽0.01. All models used splines with five degrees of freedom for the baseline hazard; the duration-dependent effects were modelled using two degrees of freedom for the TDI and HSIs, and three degrees of freedom for the PIs.

### Sample size

The expected total number of cases in the 2 areas was 265 annually. Assuming half of these could be identified prospectively from fast track and routine referrals including via colonoscopy (Barrett and Hamilton, 2008), we needed to recruit for 2 years to achieve 200 participants with CRC. We estimated one patient in 25 would have CRC (6–11% in 2-week wait clinics (Rai and Kelly, 2007), but lower out with these clinics). With 200 participants with CRC and at least 10 times as many without we would have over 80% power to detect and OR>1.52 for a common symptom occurring in half of participants. With the same 2200 patients, all of which having a TDI, we would expect to have 80% power to detect an HR>1.23 for a symptom occurring in 10% of patients.

## Results

Patients (12 236) were approached and 2667 responded, an overall 21.8% response rate (East 26.3% and North East 18.6%). Of these, 70 did not meet the eligibility criteria and a further 90 had insufficient data for analysis, leaving a final cohort of 2507 participants. The demographics of the responders were similar to those of non-responders (responders 47% male, median age 65 years; non-responders 45% male, median age 65 years). The stage distribution for those of our cohort who had CRC was comparable to 2013 data for England (late stage 50% *vs* early 40% and 10% unknown) (National Cancer Intelligence Network (NCIN)).

The characteristics of the whole cohort are provided in Tables 1 and 2. Participants were diagnosed with CRC (152, 6.1% 56% late stage), other cancers (50, 2.0%) or no cancer (2305, 91.9%). The majority of those with CRC had late-stage disease (*n*=85, 55.9% early stage: *n*=65, 42.8%); two CRCs (1.3%) were unstaged. The proportion of males was higher in the group diagnosed with CRC compared with the group diagnosed with NC (57.2% *vs* 46.2%, *P*=0.008). Those in the CRC group also had a higher median age (71 *vs* 65, *P*<0.001) and were more likely to have been referred through an urgent pathway (90.1% *vs* 71.1%, *P*<0.001). The diagnostic groups were otherwise similar in terms of deprivation, education and ethnicity (Table 2).

Among the total cohort, over half had a solitary first symptom (1332, 53.1% (CRC 92, 60.5%, NC 1218, 52.8%)). However, multiple first symptoms were common with 21.6% having two first symptoms, 9.3% having three and 8.4% with four or more multiple first symptoms. A few participants (7.6%) reported no symptoms first appearing within 2 years of diagnosis (most had symptom/s pre-dating the 2-year cut-off). Across the total cohort, change in bowel habit (62.8%) and ‘bleeding from back passage' (37.8%) were the most common symptoms, but only 45% and 24.4% of those, respectively, were reported as first symptoms (Table 3). Symptoms not specifically enquired for in the questionnaire but volunteered, such as acute gastro-intestinal illness, perianal pain, flatulence, bloating and mucus discharge, were individually reported by <3% of participants. ‘Bleeding from back passage' was significantly more frequently reported in people diagnosed with CRC than non-cancer as a first symptom (34.2% *vs* 23.9%, *P*=0.004). No other symptom as a first or subsequent symptom was more common in those with CRC than those without.

A TDI could be calculated for 2316 participants; the median TDI across the whole cohort was 136 days (IQR 74–255) (Table 4). There was no difference in the TDI between people diagnosed with CRC and NC (CRC 124 *vs* NC 138 days, *P*=0.142), and no evidence of differences between PI and HSI among those with and without CRC (PI median 41 days *vs* 36 days, *P*=0.606; HSI 49 days *vs* 59 days, *P*=0.078).

Results from the time constant survival models are shown in Table 5 (Supplementary Table A1 for univariate analyses). Older age at diagnosis and a number of symptoms were all associated with a shorter TDI, while gastrointestinal comorbidity, depression/anxiety and family history of cancer were all associated with a longer TDI and HSI.

### Symptom duration-dependent effects

We found symptom duration-dependent effects for ‘bleeding from back passage' and change in bowel habit (Supplementary Figure 1). The time-constant effects from other variables in the same model are similar to those described in the previous section and are not detailed here. The symptom ‘bleeding from back passage' was associated with briefer PI and HSI initially (HR>1), but as the duration of this symptom lengthened the association weakened (HR became close to 1). In other words, when this symptom was present it was associated with faster action (presentation to healthcare professional and/or referral) in the initial period following symptom onset or presentation, compared with other symptoms, but if initial action was not taken the existence of the symptom had little impact on action at later times. In contrast, a change in bowel habit was associated with a lengthier PI initially (HR<1) but if the symptom persisted beyond 10 days it became associated with a briefer PI (HR>1). In other words, those whose initial symptom was a change in bowel habit were less likely to present early than those with other symptoms, but of those who did not present in the first 10 days, they presented faster on average than people with other symptoms (HR>1). Change in bowel habit was always associated with a briefer HSI, with no time-dependent effect.

For early stage CRC, the median TDI for any first symptom was 157 days compared with 99 days for late stage CRC (*P*=0.019) (Table 6).

## Discussion

This is the first study worldwide to recruit a large prospective cohort of patients with suspicious symptoms before diagnosis, to study factors associated with time to diagnosis for CRC. Among these patients, who had been referred for investigation, rectal bleeding was the only symptom more frequently seen with CRC, but it occurred in only a third of cases as a first symptom and less than two-thirds at any point before diagnosis. Other so-called ‘alarm' symptoms such as change in bowel habit were also very common in this referred population but did not differentiate between CRC and those without cancer. The positive predictive value of these symptoms will be higher in this study population than in primary care, but the odds ratio between those with and without cancer will fall as this is an enriched, referred population. This does not mean these symptoms should not be taken seriously. Multiple first symptoms were common and symptoms often evolved over time before patients sought healthcare. There were different symptom and patient factors associated with the PI and HSI. Some less specific symptoms such as indigestion/abdominal pain or ‘feeling different' were associated with shorter PIs; in contrast, only the very specific, ‘classical', symptoms such as change in bowel habit and rectal bleeding were associated with shorter HSIs.

Our novel analysis of duration-dependent effects of symptoms showed different patient and healthcare provider responses to certain key symptoms of CRC according to how long the patient had, compared with other symptoms, experienced them. A short duration of rectal bleeding for some patients triggers an early consultation and prompt response by the healthcare system. Once rectal bleeding has been present for a longer time, it is no more likely than other symptoms to have a shorter diagnostic interval despite its recognition as a classical alarm symptom. This means that both patients and healthcare providers may normalise longer-term rectal bleeding and not consult or investigate promptly (Emery *et al*, 2013).

Comorbidities in the whole cohort were also associated with longer total diagnostic and healthcare intervals. Importantly, people with mental health problems, self-reported anxiety or depression, experienced a longer TDI and HSI, suggesting that their possible physical symptoms were not taken as seriously and were investigated later. Similarly, gastro-intestinal comorbidity increased time to diagnosis, probably due to healthcare providers attributing new or worsening symptoms to pre-existing illness (Emery *et al*, 2013; Hall *et al*, 2015).

There was no evidence that those with CRC were diagnosed more quickly than those with an alternative diagnosis. This is surprising given the fact that there were a higher proportion of cancers using the urgent pathway and guidance on urgent referral for suspected CRC in England recommends that ‘alarm' symptoms such as rectal bleeding and change in bowel habit warrant urgent referral for investigation (NICE, 2005). Symptoms other than rectal bleeding in this relatively large prospective cohort did not help differentiate CRC from other diagnoses.

The median PI was 35 days and median HSI (combining primary and secondary care) was 58 days. This contrasts with findings of the secondary analysis of CRC data in the National Audit of Cancer Diagnosis in Primary Care, where data from primary care medical records showed a median PI of 19 days (Lyratzopoulos *et al*, 2015b). In a separate study using the same data set, 21% of CRC patients had three or more primary care consultations before referral, potentially contributing to longer HSIs (Lyratzopoulos *et al*, 2013). However, in an analysis of UK General Practice Research Database data for 2962 patients with CRC in 2007/8, the median HSI, of 80 days, was more comparable to our own findings (Neal *et al*, 2014a).

Key strengths of this study were the prospective design and the collection of data from several sources: patient reports and primary care and specialist records. The analytical and reporting approaches were robust and performed according to the methodological approaches and definitions recommended in the Aarhus statement (Weller *et al*, 2012) and STROBE guidelines (von Elm *et al*, 2007). We chose to define the date of first symptom/s using the patient-reported date rather than the primary care-reported date, as we were analysing patient-reported symptom/s. Ideally, a study would recruit patients from primary care before referral; however, this would be accompanied by major logistical and resource implications in identifying an extremely large prospective cohort of patients with colorectal symptoms, to capture sufficient patients with cancer. Instead, we recruited patients when they first encountered secondary care; this had the added benefit of allowing us to recruit patients presenting as emergencies and those referred from other specialists. Recruitment involved two regions of England, selected to ensure a broad range of socio-economic, educational and occupational levels, and the deprivation data suggest that the cohort was reasonably representative of the national population.

The main study limitation is the overall recruitment rate of 22%, although this is similar to other recent studies (Kidney *et al*, 2015; Walter *et al*, 2015). We sought to make contact with the target population before they underwent investigation and received a diagnosis, and it is possible that some were unable or unwilling to complete a questionnaire at this worrying time. We are also likely to have under-recruited people who presented as an emergency or who died soon after presentation. However, a recent study from the English Cancer Patient Experience Study showed that only 6% of eligible patients died between sampling and mail-out, suggesting potential survival bias in these types of study is relatively small (Abel *et al*, 2016). The demographics of non-responders were very similar to participants and the proportion of late-stage CRC was comparable to national data, suggesting our cohort was reasonably representative, that selection bias was not a major issue, and that the findings can be generalised to similar symptomatic populations. If sicker patients were less likely to take part in the study and they were more likely to have shorter intervals, we may have overestimated the typical intervals in the population. We may well have also underestimated differences between those with different presenting symptoms. In common with a Danish prospective population-based study of diagnostic intervals, the problem of confounding by indication remains to some extent (Torring *et al*, 2012). Our results did not find that shorter time to diagnosis was associated with earlier stage disease. It may be that late-stage disease has different symptom profiles, which affect help-seeking and use of diagnostic pathways. Tumour factors (such as histological type, rate of growth and location) and host factors (such as comorbidity) can influence diagnostic intervals and result in apparently earlier diagnosis of later stage disease. As our study set out to investigate the diagnostic intervals from perception of first symptom to diagnosis, our primary exposure was the initial symptom or symptoms. Although this approach allows us to make robust comparisons with other findings, it may obscure the finer detail of symptom patterns and clusters as they evolve over time and their effects on timely help-seeking by patients and timely diagnosis in primary and secondary care. Although this was a large cohort, we had insufficient power to examine specific clusters of symptoms and their associations with outcomes; a much larger prospective study would be required to achieve this. The fact that fewer CRC cases were recruited than was the aim meant that the study was only powered to detect large differences in symptoms between those with and without CRC.

This study shows that there are subtle differences in the impact of symptoms and patient factors on patient and healthcare intervals, with some clear implications for policy makers and clinicians. Although rectal bleeding was the only symptom predictive of CRC in this referred population, it was only reported as the first symptom in one-third of cases and as a subsequent symptom in a further 25% of cases. Despite conducting such a large prospective cohort study, we failed to identify any other strong solitary symptom signals of CRC, suggesting that bowel cancer awareness campaigns, which currently concentrate on a single symptom, should also consider messages that reflect the importance of multiple symptoms and evolution of symptoms over time (Moffat *et al*, 2015). The recently revised NICE guidelines for early detection of CRC support this premise and have also lowered the threshold for referral, in line with patient preferences for investigation (Banks *et al*, 2014a, 2014b). However, our study has also shown that only people presenting with shorter histories of rectal bleeding are investigated promptly, and that healthcare professionals should remain alert to symptoms of possible CRC in people with a history of gastro-intestinal or mental health conditions. The increasingly widespread use of clinical decision support in primary care can also be informed by our findings (Dikomitis *et al*, 2015; Green *et al*, 2015), but further research is needed, alongside GPs and specialists, to identify mechanisms by which patients can be identified, referred and diagnosed in the most timely and appropriate way.

In conclusion, as efforts to expedite the diagnosis of symptomatic CRC are likely to have benefits for patients in terms of improved survival, earlier-stage diagnosis and improved quality of life, it continues to be a priority to identify symptoms and other factors, which should prompt an individual to seek help or a GP to refer in an appropriate and timely manner. It is also important to develop other strategies for earlier diagnosis, including increasing uptake of CRC screening and perhaps the development of biomarkers to improve early detection. Nevertheless, these data provide support for more targeted evaluation of suspicious symptoms in an attempt to identify CRC at an earlier and more amenable stage. It may also be that targeted interventions at higher risk populations aimed at symptom monitoring could be more effective at recognising symptom evolution.

## Figures and Tables

**Table 1:** Diagnostic characteristics of participants

	**Total (%)**
**Primary CRC**	152 (6.1)
**Other cancers**	50 (2.0)
Lymphoma	9
Prostate cancer	7
Gynaecological cancer (ovarian, uterine)	7
Anal cancer	5
Renal cancer (kidney, bladder)	4
Cancer unknown primary	4
Oesophageal cancer	3
Pancreatic cancer	2
Multiple myeloma	2
Stomach cancer	2
Other^a^	5
**Non-cancer**^a^	2305 (91.9)
Polyps	612 (26.6)
Polyp with metaplasia	200 (8.7)
Poly with dysplasia	168 (7.3)
Polyp NOS	244 (10.6)
Nil abnormal	598 (25.9)
Diverticular disease	463 (20.1)
Inflammatory bowel disease (IBD)	189 (8.2)
Colitis ulcerative	55 (2.4)
Colitis–other	99 (4.3)
Crohn's disease	7 (0.3)
IBD NOS	28 (1.2)
Haemorrhoids	186 (8.1)
Irritable bowel syndrome	95 (4.1)
Benign gastro-duodenal disease	83 (3.6)
Miscellaneous	51 (2.2)
Anaemia (no lower GI cause found)	31 (1.3)
Hepato-pancreato-biliary disorders	20 (0.9)
Anal conditions (excluding haemorrhoids)	13 (0.6)
Constipation	13 (0.6)
Coeliac disease	5 (0.2)
Total	2507

Abbreviations: CRC=colorectal cancer; GI=gastrointestinal; NOS=not otherwise specified.

Other cancers include (*n*=1): leiomyosarcoma, appendix, peritoneal, melanoma, cholangiocarcinoma.

a Percentages add to >100%, because 54 patients have more than one diagnosis. More than one diagnosis in the same category (e.g., miscellaneous) was counted only once.

**Table 2:** Socio-demographic characteristics of participants

	**CRC (*****n*****=152)**	**OC (*****n*****=50)**	**NC (*****n*****=2305)**	**Total cohort** (***n*****=2507)**	***P*****-value** **CRC** ***vs*** **NC**
Age (median, range)	71 (40–92)	71.5 (51–91)	65 (40–100)	65 (40–100)	<0.001
**Gender**					
Male	87 (57.2)	30 (60)	1064 (46.2)	1181 (47.1)	0.008
Female	65 (42.8)	20 (40)	1241 (53.8)	1326 (52.9)	
**Highest education level**					
Degree/diploma/equivalent	67 (44.1)	17 (34.0)	941 (40.8)	1025 (40.9)	0.511
A level/GCSE/O level	42 (27.6)	16 (32.0)	740 (32.1)	798 (31.8)	
Other/none/missing	43 (28.3)	17 (34.0)	624 (27.1)	684 (27.3)	
**Ethnicity**					
White	149 (98.0)	50 (100)	2259 (98.0)	2458 (98.1)	0.640
Other/missing	3 (2.0)	0	46 (2.0)	49 (2.0)	
**Smoking status**					
Current	16 (10.5)	2 (4.0)	205 (8.9)	223 (8.9)	0.549
Ex-smoker	68 (44.7)	21 (42.0)	970 (42.1)	1059 (42.2)	
Never/missing	68 (44.7)	27 (54.0)	1130 (49.0)	1225 (48.9)	
**Lives alone**					
Yes	43 (28.3)	14 (28.0)	538 (23.3)	595 (23.7)	0.164
No/missing	109 (71.8)	36 (72.0)	1767 (76.7)	1912 (76.3)	
**Deprivation (IMD quintiles)**					
First national quintile (least)	61 (40.1)	13 (26.0)	826 (35.8)	900 (35.9)	0.168
Second national quintile	40 (26.3)	12 (24.0)	550 (23.9)	602 (24.0)	
Third national quintile	12 (7.9)	10 (20.0)	352 (15.3)	374 (14.9)	
Fourth national quintile	20 (13.2)	10 (20.0)	304 (13.2)	334 (13.3)	
Fifth national quintile (most)	19 (12.5)	5 (10.0)	263 (11.4)	287 (11.4)	
missing	0	0	10 (0.4)	10 (0.4)	

Abbreviations: CRC=colorectal cancer; IMD=Index of Multiple Deprivation; OC=other cancer; NC=no cancer.

Values are *n* (%), unless otherwise stated. Missing values were included in the Other/Never categories where possible. Percentages are as follows: Employment Status (2.0), Education Level (3.3), Ethnicity (0.8), Smoking Status (1.5), Live Alone (0.6), referral route (2.2).

**Table 3:** Symptoms reported by participants, stratified by all and first symptom/s and diagnostic group

	**CRC (*****n*****=152)**		**NC (*****n*****=2305)**		**Total cohort (*****n*****=2507)**		
**Symptom**	**First symptoms**	**Subsequent symptoms**	**First symptoms**	**Subsequent symptoms**	**First symptoms**	**Subsequent symptoms**	***P*****-value for first symptoms CRC** ***vs*** **NC**
**Symptoms in questionnaire**							
Change in bowel habit	66 (43.4)	27 (17.8)	1043 (45.2)	410 (17.8)	1129 (45)	446 (17.8)	0.661
‘Bleeding from back passage'	52 (34.2)	39 (25.7)	552 (23.9)	296 (12.8)	612 (24.4)	336 (13.4)	0.004
Indigestion/heartburn/persistent tummyache	30 (19.7)	13 (8.6)	509 (22.1)	281 (12.2)	549 (21.9)	302 (12)	0.499
Fatigue or tiredness that is unusual for you	35 (23)	23 (15.1)	404 (17.5)	307 (13.3)	448 (17.9)	347 (13.8)	0.086
Feeling different ‘in yourself' from usual	27 (17.8)	11 (7.2)	391 (17)	257 (11.1)	433 (17.3)	278 (11.1)	0.799
Decrease in appetite	15 (9.9)	16 (10.5)	195 (8.5)	192 (8.3)	220 (8.8)	221 (8.8)	0.547
Back pain	3 (2)	9 (5.9)	197 (8.5)	179 (7.8)	210 (8.4)	194 (7.7)	0.004
Unexplained weight loss	6 (3.9)	18 (11.8)	111 (4.8)	183 (7.9)	127 (5.1)	214 (8.5)	0.626
**Other symptoms reported by participants**							
Other (non-gastro-intestinal) symptoms	4 (2.6)	3 (2)	47 (2)	16 (0.7)	53 (2.1)	20 (0.8)	NA
Acute gastro-intestinal illness	0 (0)	3 (2)	48 (2.1)	15 (0.7)	49 (2)	18 (0.7)	NA
Perianal pain or discomfort	2 (1.3)	1 (0.7)	37 (1.6)	15 (0.7)	39 (1.6)	16 (0.6)	NA
Wind or flatulence	0 (0)	2 (1.3)	33 (1.4)	11 (0.5)	33 (1.3)	13 (0.5)	NA
Urgency or leakage of bowels	0 (0)	0	31 (1.3)	13 (0.6)	32 (1.3)	13 (0.5)	NA
Bloating	0 (0)	1 (0.7)	26 (1.1)	12 (0.5)	28 (1.1)	14 (0.6)	NA
Mucus or discharge per rectum	0 (0)	2 (1.3)	25 (1.1)	7 (0.3)	25 (1)	9 (0.4)	NA
Pain, non-abdominal	1 (0.7)	0	22 (1)	9 (0.4)	23 (0.9)	9 (0.4)	NA

Abbreviations: CRC=colorectal cancer; NA=not applicable; NC=no cancer.

Values are *n* (%), unless otherwise stated, and all columns add up to >100% because of multiple symptoms.

**Table 4:** TDI, PI and HSI for first symptom/s for total cohort and by diagnostic groups

	**Total cohort**		**CRC**		**NC**	
**Symptom**	**Median (IQR)**	***n***	**Median (IQR)**	***n***	**Median (IQR)**	***n***
**(A) TDI**^a^						
Any symptom	136 (74–255)	2316	124 (74–226)	149	138 (74–264)	2120
Change in bowel habit	113 (69–203)	1129	124 (66–226)	66	112 (70–203)	1043
‘Bleeding from back passage'	90 (49–164)	612	88 (47–142)	52	89 (49–173)	552
Indigestion/heartburn/persistent tummy ache	139 (81–277)	549	125 (80–262)	30	142 (81–283)	509
Fatigue or tiredness that is unusual for you	143 (86–239)	448	130 (92–189)	35	146 (86–247)	404
Feeling different ‘in yourself' from usual	128 (77–216)	433	124 (80–154)	27	130 (76–231)	391
Decrease in appetite	115 (69–235)	220	98 (64–138)	15	115 (69–269)	195
Back pain	161 (79–294)	210	98 (92–525)	3	162 (79–295)	197
Unexplained weight loss	148 (84–333)	127	111 (82–205)	6	159 (86–346)	111
**(B) PI**^b^						
Any symptom	35 (7–92)	2103	41 (13–92)	128	36 (7–93)	1932
Change in bowel habit	42 (14–92)	1106	57 (22–115)	63	42 (14–92)	1023
‘Bleeding from back passage'	25 (3–69)	607	29 (6–66)	52	24 (3–69)	547
Indigestion/heartburn/persistent tummy ache	31 (5–86)	525	28 (7–90)	27	31 (5–90)	488
Fatigue or tiredness that is unusual for you	31 (7–89)	365	35 (8–65)	26	31 (6–90)	331
Feeling different ‘in yourself' from usual	30 (4–76)	377	37 (14–65)	21	30 (3–77)	343
Decrease in appetite	20 (3–62)	190	33 (8–90)	15	18 (2–62)	166
Back pain	29 (2–76)	183	41 (37–64)	3	29 (1–88)	172
Unexplained weight loss	31 (5–92)	115	46 (35–73)	4	31 (2–96)	102
**(C) HCI**^b^						
Any symptom	58 (27–128)	2103	49 (26–106)	128	59 (27–129)	1932
Change in bowel habit	51 (25–99)	1106	47 (28–101)	63	52 (25–99)	1023
‘Bleeding from back passage'	41 (19–85)	607	37 (20–60)	52	41 (19–86)	547
Indigestion/heartburn/persistent tummy ache	76 (34–156)	525	58 (35–151)	27	78 (34–161)	488
Fatigue or tiredness that is unusual for you	69 (30–146)	365	54 (27–89)	26	70 (29–147)	331
Feeling different ‘in yourself' from usual	67 (32–139)	377	61 (38–87)	21	69 (30–144)	343
Decrease in appetite	60 (31–138)	190	49 (30–63)	15	64 (30–158)	166
Back pain	88 (35–203)	183	61 (51–461)	3	82 (35–206)	172
Unexplained weight loss	83 (39–191)	115	33 (16–50)	4	89 (43–202)	102

Abbreviations: CRC=colorectal cancer; HCI=healthcare interval; HSI=health system interval; IQR, interquartile range; NC=no cancer; PI=patient interval; TDI=total diagnostic interval.

*P*-values for CC *vs* NC with any symptom: (A) TDI *P*=0.142; (B) PI *P*=0.606; (C) HSI *P*=0.078.

Similar results were obtained for the comparison between all cancers and NC (data not shown).

a As these are median time intervals (rather than mean intervals), there is no expectation that the median TDI will equal the sum of median PI and median HSI.

b The PI and HSI could only be calculated for 2103 participants (unknown presentation date *n*=213). Those with an available date of presentation had a shorter median TDI (130 days *vs* 199 days for the remaining cases).

**Table 5:** Predictors of TDI, PI and HSI

	**TDI (*****n*****=2306)**		**PI (*****n*****=2095)**		**HSI (*****n*****=2095)**	
	**HR (95% CI)**	***P*****-value**	**HR (95% CI)**	***P*****-value**	**HR (95% CI)**	***P*****-value**
Bleeding from back passage	1.79 (1.62–1.98)	<0.001	1.41 (1.27–1.57)	<0.001	1.56 (1.41–1.72)	<0.001
Change in bowel habit	1.55 (1.42–1.69)	<0.001	1.04 (0.95–1.13)	0.440	1.55 (1.42–1.70)	<0.001
Back pain	0.99 (0.85–1.14)	0.874	1.20 (1.02–1.40)	0.024	0.86 (0.73–1.00)	0.052
Indigestion/heartburn/tummy ache	1.00 (0.90–1.10)	0.942	1.17 (1.06–1.30)	0.003	0.84 (0.76–0.93)	0.001
Decrease in appetite	1.10 (0.95–1.28)	0.200	1.15 (0.98–1.35)	0.087	1.11 (0.94–1.30)	0.224
Weight loss	0.90 (0.75–1.09)	0.284	1.00 (0.82–1.22)	0.987	0.81 (0.66–0.99)	0.038
Fatigue or tiredness	1.14 (1.02–1.27)	0.021	1.11 (0.98–1.25)	0.096	1.04 (0.92–1.17)	0.549
Feeling different	1.21 (1.08–1.35)	0.001	1.25 (1.10–1.41)	<0.001	1.01 (0.90–1.14)	0.812
Gender (ref=female)						
Male	1.01 (0.93–1.10)	0.739	1.03 (0.94–1.13)	0.535	1.02 (0.93–1.11)	0.731
Age at diagnosis (10 years)	1.08 (1.04–1.12)	<0.001	1.13 (1.08–1.18)	<0.001	0.99 (0.95–1.03)	0.698
IMD (ref=least deprived)		0.611		0.645		0.528
Second national quintile	0.98 (0.88–1.09)		1.03 (0.91–1.15)		0.92 (0.82–1.03)	
Third national quintile	1.03 (0.90–1.17)		1.09 (0.95–1.25)		0.94 (0.82–1.08)	
Fourth national quintile	0.91 (0.79–1.04)		0.97 (0.84–1.12)		0.90 (0.78–1.04)	
Fifth national quintile	0.97 (0.83–1.13)		0.98 (0.83–1.16)		0.96 (0.81–1.13)	
Gastrointestinal comorbidity	0.80 (0.71–0.89)	<0.001	1.01 (0.89–1.14)	0.904	0.78 (0.69–0.88)	<0.001
Depression/anxiety	0.86 (0.77–0.96)	0.007	1.05 (0.93–1.18)	0.415	0.80 (0.71–0.90)	<0.001
Family History of cancer	0.91 (0.83–0.99)	0.031	0.94 (0.86–1.04)	0.237	0.90 (0.82–0.99)	0.033

Abbreviations: CI=confidence interval; HSI=health system interval; HR=hazard ratio; PI=patient interval; TDI=total diagnostic interval.

HR estimates are from a flexible parametric survival multivariable model with only time constant effects. Model is adjusted for all variables in table plus ethnicity (non-White *vs* White), smoking status (current and ex-smoker *vs* never), living alone (yes *vs* no) and region (North East *vs* Cambridge). In this context, the HR represents the relative increase in rate of presentation/diagnosis. A HR of 2 would imply that patients in one group presented/were diagnosed twice as quickly as in the reference group.

**Table 6:** Time to diagnosis (days) for first symptom/s among CRC group, stratified by stage at diagnosis^a^

	**Early stage (*****n*****=64)**		**Late stage (*****n*****=82)**	
	**Median (IQR)**	***n***	**Median (IQR)**	***n***
Any symptom	157 (93–243)	64	99 (65–198)	83
Change in bowel habit	159 (92–274)	23	111 (65–173)	41
‘Bleeding from back passage'	92 (36–156)	23	84 (47–133)	28
Indigestion or heartburn or persistent tummy ache that wasn't normal for you	188 (96–310)	11	103 (62–153)	18
Fatigue or tiredness that is unusual for you	154 (95–226)	15	110 (81–152)	20
Feeling different ‘in yourself' from usual	114 (95–154)	6	101 (65–153)	18
Decrease in appetite	138 (95–186)	3	83 (56–128)	11
Back pain	–	0	98 (92–525)	3
Unexplained weight loss	205 (82–572)	3	69 (39–98)	2

Abbreviations: IQR=interquartilerange.

a Not staged: 2. *P*-values for early *vs* late stage with any symptom: (i) *P*=0.019, (ii) *P*=0.022.
