# Supplementary material for: Symptoms and patient factors associated with longer time to diagnosis for colorectal cancer: results from a prospective cohort study
Source: Br J Cancer. 2016 Aug 4;115(5):533–41. doi: 10.1038/bjc.2016.221 (PMC4997546; doi:10.1038/bjc.2016.221)

**SUPPLEMENTARY ONLINE MATERIAL**

**Figure 1: Time dependent effects of ‘bleeding from back passage’ and ‘change in bowel habit’ on the hazard ratio (HR) of Total Diagnostic Interval (TDI), Patient Interval (PI) and Health System Interval (HSI)**


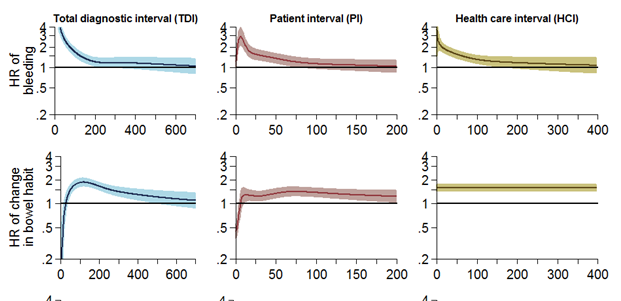

Supplement: Supplementary Figure 1 [file bjc2016221x1.doc]
