# Supplementary material for: Symptoms and patient factors associated with longer time to diagnosis for colorectal cancer: results from a prospective cohort study
Source: Br J Cancer. 2016 Aug 4;115(5):533–41. doi: 10.1038/bjc.2016.221 (PMC4997546; doi:10.1038/bjc.2016.221)
Supplement: Supplementary Table 1 [file bjc2016221x2.doc]

**SUPPLEMENTARY ONLINE MATERIAL**

**Table 1: Univariable analysis of factors associated with Total Diagnostic Interval (TDI), Patient Interval (PI) and Health System Interval (HSI)**

Hazard ratio estimates are from flexible parametric survival univariate models.

|  | **Total Diagnostic Interval**  **N=2316** | | **Patient Interval**  **N=2103** | | **Health System Interval**  **N=2103** | |
| --- | --- | --- | --- | --- | --- | --- |
|  | **HR (95 CI)** | **p-value** | **HR (95 CI)** | **p-value** | **HR (95 CI)** | **p-value** |
| **‘Bleeding from Back Passage’** | 1.59 (1.45-1.75) | <0.001 | 1.25 (1.14-1.37) | <0.001 | 1.52 (1.38-1.67) | <0.001 |
| **Change in Bowel Habit** | 1.43 (1.32-1.55) | <0.001 | 0.97 (0.89-1.05) | 0.424 | 1.46 (1.34-1.60) | <0.001 |
| **Back pain** | 0.88 (0.76-1.02) | 0.081 | 1.21 (1.04-1.41) | 0.015 | 0.77 (0.66-0.89) | 0.001 |
| **Indigestion/Heartburn/Tummy Ache** | 0.92 (0.84-1.01) | 0.091 | 1.13 (1.02-1.24) | 0.017 | 0.78 (0.71-0.86) | <0.001 |
| **Decrease in Appetite** | 1.07 (0.93-1.23) | 0.322 | 1.33 (1.14-1.54) | <0.001 | 0.99 (0.86-1.15) | 0.932 |
| **Weight loss** | 0.83 (0.70-1.00) | 0.047 | 1.03 (0.86-1.25) | 0.726 | 0.79 (0.66-0.95) | 0.015 |
| **Fatigue or Tiredness** | 0.99 (0.89-1.10) | 0.833 | 1.13 (1.01-1.27) | 0.032 | 0.91 (0.82-1.02) | 0.119 |
| **Feeling Different** | 1.14 (1.02-1.26) | 0.017 | 1.30 (1.16-1.45) | <0.001 | 0.97 (0.86-1.08) | 0.538 |
| **Gender** (ref=Female) |  |  |  |  |  |  |
| Male | 1.10 (1.01-1.19) | 0.027 | 1.04 (0.95-1.13) | 0.367 | 1.08 (0.99-1.18) | 0.083 |
| **Age at diagnosis (10 years)** | 1.06 (1.02-1.10) | 0.001 | 1.10 (1.06-1.14) | <0.001 | 1.01 (0.98-1.05) | 0.527 |
| **Ethnicity** (ref= White) |  |  |  |  |  |  |
| Other | 0.75 (0.55-1.04) | 0.083 | 0.83 (0.59-1.16) | 0.270 | 0.75 (0.53-1.05) | 0.090 |
| **Smoking Status** (ref= Never) |  | 0.702 |  | 0.236 |  | 0.292 |
| Current | 0.94 (0.81-1.10) |  | 1.05 (0.90-1.23) |  | 0.94 (0.81-1.10) |  |
| Ex-Smoker | 1.01 (0.92-1.10) |  | 0.94 (0.86-1.03) |  | 1.05 (0.96-1.15) |  |
| **Live Alone** (ref= No) |  |  |  |  |  |  |
| Yes | 0.97 (0.88-1.06) | 0.468 | 1.05 (0.95-1.17) | 0.306 | 0.92 (0.83-1.02) | 0.098 |
| **IMD** (ref=least deprived) |  | 0.644 |  | 0.334 |  | 0.068 |
| 2^nd^ National Quintile | 1.00 (0.90-1.12) |  | 1.05 (0.93-1.17) |  | 1.00 (0.89-1.11) |  |
| 3^rd^ National Quintile | 1.04 (0.92-1.18) |  | 1.15 (1.01-1.31) |  | 1.02 (0.89-1.16) |  |
| 4^th^ National Quintile | 1.04 (0.91-1.18) |  | 1.02 (0.89-1.16) |  | 1.06 (0.93-1.22) |  |
| 5^th^ National Quintile | 1.11 (0.96-1.27) |  | 1.06 (0.92-1.23) |  | 1.23 (1.06-1.42) |  |
| **GI Comorbidity** | 0.76 (0.68-0.85) | <0.001 | 1.05 (0.93-1.18) | 0.442 | 0.73 (0.65-0.82) | <0.001 |
| **Depression/anxiety** | 0.83 (0.75-0.93) | 0.001 | 1.03 (0.92-1.16) | 0.560 | 0.79 (0.70-0.88) | <0.001 |
| **Family History of cancer** | 0.89 (0.82-0.97) | 0.011 | 0.94 (0.85-1.03) | 0.157 | 0.93 (0.84-1.01) | 0.098 |
| **Region** (ref=Cambridge) |  |  |  |  |  |  |
| **North East** | 1.33 (1.22-1.44) | <0.001 | 1.08 (0.99-1.17) | 0.093 | 1.51 (1.38-1.64) | <0.001 |
