# Supplementary material for: Symptoms and patient factors associated with longer time to diagnosis for colorectal cancer: results from a prospective cohort study
Source: Br J Cancer. 2016 Aug 4;115(5):533–41. doi: 10.1038/bjc.2016.221 (PMC4997546; doi:10.1038/bjc.2016.221)
Supplement: Supplementary Appendix [file bjc2016221x3.docx]

APPENDIX A

**Inclusion–exclusion recruitment criteria**

(i) Fast/urgent clinic track referrals and emergency admissions: include ALL.

(ii) Routine referrals (and via colonoscopy): include patients with

- Change in bowel habit (including diarrhoea or constipation)
- Rectal bleeding/blood in stool
- Abdominal pain (in presence of weight loss)
- Abdominal mass
- Anaemia
- Expressed concern about possibility of cancer

Exclude:

- Review of established diagnosis where there is no cancer concern
- Previous GI cancer
- Known infective diarrhoea (e.g., after recent foreign travel)
- Abdominal pain in the absence of weight loss

APPENDIX B

The questionnaire starts with: ‘We are interested in symptoms which you think are related to your recent referral to hospital. The following questions are about when you first noticed a symptom and when you first told your GP or nurse about it. Please give an exact date if you can. Otherwise, please give your best estimate (for example, approximately how long ago, the month or the season). You may wish to refer to your diary or calendar if you have it with you’. The first question was: ‘What was the first thing or symptom you noticed that made you think something might be wrong?’ Eight specific symptoms followed: change in bowel habit; ‘bleeding from the back passage’; back pain; indigestion or abdominal pain ‘that wasn’t normal for you’; plus decrease in appetite, unexplained weight loss, fatigue or tiredness ‘that is unusual for you’, and feeling different ‘in yourself’ from usual.
